# High diversity of clinical *Mycobacterium intracellulare* in China revealed by whole genome sequencing

**DOI:** 10.3389/fpubh.2022.989587

**Published:** 2022-11-17

**Authors:** Zexuan Song, Zhi Liu, Aijing Ma, Chunfa Liu, Wencong He, Xiangjie Zeng, Yiting Wang, Ping He, Dongxin Liu, Bing Zhao, Hui Xia, Shengfen Wang, Yanlin Zhao

**Affiliations:** ^1^National Institute for Communicable Disease Control and Prevention, Chinese Center for Disease Control and Prevention, Beijing, China; ^2^Department of the Third Pulmonary Disease, Shenzhen Third People's Hospital of Shenzhen, Shenzhen, China; ^3^National Tuberculosis Reference Laboratory, Chinese Center for Disease Control and Prevention, Beijing, China; ^4^Institute for Communicable Disease Control and Prevention, Hainan Centre for Disease Control and Prevention, Haikou, China; ^5^National Clinical Research Centre for Infectious Diseases, Shenzhen Third People's Hospital, Shenzhen, China

**Keywords:** *Mycobacterium intracellulare*, drug resistance profile, nontuberculous mycobacterial lung disease, *Mycobacterium indicus prani*, whole genome sequencing

## Abstract

*Mycobacterium intracellulare* is the most common cause of nontuberculous mycobacterial lung disease, with a rapidly growing prevalence worldwide. In this study, we performed comparative genomic analysis and antimicrobial susceptibility characteristics analysis of 117 clinical *M. intracellulare* strains in China. Phylogenetic analysis showed that clinical *M. intracellulare* strains had high genetic diversity and were not related to the geographical area. Notably, most strains (76.07%, 89/117) belonged to *Mycobacterium paraintracellulare* (MP) and *Mycobacterium indicus pranii* (MIP) in the genome, and we named them MP-MIP strains. These MP-MIP strains may be regarded as a causative agent of chronic lung disease. Furthermore, our data demonstrated that clarithromycin, amikacin, and rifabutin showed strong antimicrobial activity against both *M. intracellulare* and MP-MIP strains *in vitro*. Our findings also showed that there was no clear correlation between the *rrs, rrl*, and DNA gyrase genes (*gyrA* and *gyrB*) and the aminoglycosides, macrolides, and moxifloxacin resistance, respectively. In conclusion, this study highlights the high diversity of *M. intracellulare* in the clinical setting and suggests paying great attention to the lung disease caused by MP-MIP.

## Introduction

*Mycobacterium intracellulare* (*M. intracellulare*), a major species of the *Mycobacterium avium* complex (MAC), is the leading cause of nontuberculous mycobacterial lung disease worldwide (1, 2). It can cause lung illness in both immunocompetent and immunosuppressed patients, showing common respiratory symptoms such as cough, sputum, and weight loss (3). *M. intracellulare* is ubiquitous in the environment, such as in water and soil. Some studies showed that residential environments like bathroom or drinking water could be the sources of infection (4). In recent years, the incidence of *M. intracellulare* infections is growing, causing widespread concern and attention (2). Researchers in Korea retrospectively investigated data on *Mycobacterium species* over 13 years in their country, showing that the most common species was *M. intracellulare* (50.6%) (5). A national survey of nontuberculous mycobacteria pulmonary disease in China showed that 34.1% of the strains belong to MAC, of which *M. intracellulare* is the most common and distributed widely (6). Thus, accurate identification of the *M. intracellulare* from patients and timely treatment are particularly important.

*Mycobacterium indicus pranii* (MIP) has been considered a non-pathogenic and cultivable organism with immunomodulatory characteristics, which has therapeutic value in the treatment of leprosy (7, 8). Its taxonomic characterization showed high sequence identity (>99%) to *M. intracellulare* based on the most common housekeeping genes as well as similar phenotypic characteristics, such as the negative urease (9). *Mycobacterium yongonense* and *Mycobacterium chimaera* are opportunistic pathogens, which could cause pulmonary infections in humans, usually in immunocompromised patients and in patients with underlying respiratory diseases (10, 11). To date, MIP, *Mycobacterium yongonense* and *Mycobacterium chimaera* have been regarded as *M. intracellulare subsp. intracellulare* (12)*, Mycobacterium intracellulare subsp. yongonense* and *Mycobacterium intracellulare subsp. chimaera*, respectively, being the subspecies of *M. intracellulare*. *Mycobacterium paraintracellulare* (MP) is an independent species in the NCBI database, but previous reports showed that *M. paraintracellulare* should be reclassified into *M. intracellulare* at the subspecies level with high sequence similarity (average nucleotide identity ≥98%) (13, 14).

Lately, some studies have revealed that MIP could cause NTM pulmonary disease in clinical trials, and some strains were misdiagnosed as *M. intracellulare* because of the high similarity in clinical diagnosis (15, 16). A recent study about the genomic analysis of *M. intracellulare* and related species isolates showed that clinical *M. intracellulare* strains have been separated into two major groups: the typical *M. intracellulare* (TMI) group and the *M. paraintracellulare*- *M. indicus pranii* (MP-MIP) group (12). Thus, MIP should be considered a cause of pulmonary disease in humans with pre-existing lung diseases, such as tuberculosis and bronchiectasis (16).

Antimicrobial susceptibility information is considered critical for the successful and appropriate treatment of pulmonary illnesses (17, 18). Official clinical practice guidelines suggest that macrolide, ethambutol, and rifamycin (or rifabutin) should be included in treatment regimens for MAC infections, and amikacin or streptomycin may be added to the treatment regimens if the patient is macrolide-resistant or requires more aggressive therapy (18). However, the subspecies of MAC strains exhibit various drug susceptibility patterns. Researchers have investigated the differences in drug susceptibility of the subspecies strains, such as *M. intracellulare, Mycobacterium avium, Mycobacterium intracellulare subsp. chimaera*, and *Mycobacterium colombiense* (19, 20), but there is little information available regarding the drug susceptibility of MP and MIP.

In this study, we compared the genomics of 117 clinical strains that were previously identified with *M. intracellulare* to comprehend the genetic diversity and similarities of clinically isolated *M. intracellulare* strains in China. In addition, we further investigated the antimicrobial susceptibility characteristics of the strains, especially for the MP and MIP, which could increase the body of available MIC data and provide the basis for clinical treatment.

## Materials and methods

### Isolates collection and identification

A total of 117 clinical strains that were previously identified as *M. intracellulare* were randomly selected from the nontuberculous mycobacteria database of the national tuberculosis reference laboratory in China (6). The species of nontuberculous mycobacteria strains were identified by MALDI-TOF MS after four weeks grown on Lowenstein Jensen media and sequencing their 16S ribosomal genes. In this study, the subspecies were confirmed by the average nucleotide identity (ANI) and phylogenetic analysis based on whole genome sequence (WGS). The ANI was calculated by an ANI Calculator online (https://www.ezbiocloud.net/tools/ani). The pairwise ANI values were determined by pyani (https://github.com/widdowquinn/pyani) and visualized using the heatmap of the R package. The *M. intracellulare* ATCC 13950 (NC_016946.1), MIP MTCC 9506 (NC_018612.1), *M. intracellulare subsp. yongonense* 05-1390 (NC_021715.1), *Mycobacterium paraintracellulare* MOTT64 (NC_016948.1), and *M. intracellulare subsp. chimaera* DSM 44623(NZ_CP015278.1) were set as the subspecies reference genomes.

### Antimicrobial susceptibility testing

According to the Clinical and Laboratory Standards Institute (CLSI) standard guideline, the antimicrobial susceptibility testing in this study was performed using the Sensititre™ SLOWMYCOI panel (21). It included 13 antimicrobials: clarithromycin (CLR), amikacin (AN), moxifloxacin (MXF), linezolid (LNZ), ciprofloxacin (CIP), doxycycline (DO), ethambutol (EMB), rifampicin (RIF), rifabutin (RFB), sulfamethoxazole (SXT), ethionamide (ETH), isoniazid (INH), and streptomycin (SM). The resistance breakpoints were determined as previously described according to CLSI standards (6).

### DNA extraction and sequencing

The 117 strains were cultured on Lowenstein Jensen media and genome DNA was extracted following the protocol of the cetyltrimethylammonium bromide (CTAB) method (22). Whole-genome sequencing was performed on the Illumina Hiseq PE150 platform by Annoroad (Beijing, China). The paired-end reads were examined using FastQC (v0.11.9) and trimmed using Trimmomatic (v0.39) (23). The genome sequences were assembled into a number of scaffolds by SPAdes (24). And the quality of the assemblies was evaluated using QUAST (v5.0.2).

### Phylogenetic analysis

To get a better understanding of the population structure of *M. intracellulare*, we downloaded the publicly available genomes of 30 *M. intracellulare*, 2 MIP, 3 *Mycobacterium intracellulare subsp. Yongonense* and 9 *Mycobacterium paraintracellulare* from the NCBI public database (Supplementary Table S1). Single nucleotide polymorphisms (SNPs) were extracted by snippy pipeline (v4.3.6) with the reference genome ATCC 13950 (NC_016946.1) (https://github.com/tseemann/snippy). The maximum-likelihood phylogenetic tree based on the core SNPs was constructed by RAxML-NG, using 1000 bootstrap iterations and the GTR+G model. The genome comparison of the two strains was calculated by the ANI online tools (https://www.ezbiocloud.net/tools) (25).

### Statistical analysis

All statistical analyses were performed by SPSS v18.0 software (SPSS Inc. USA), Chi-square test or Fisher exact test was used for categorical data. *P* < 0.05 was considered statistically significant.

## Results

### The species re-identification

In our study, 117 clinical *M. intracellulare* strains have been re-identified by the average nucleotide identity (ANI) of the whole genome sequence, and the strains are separated into two major groups (Supplementary Figure S1). The reference strains *M. intracellulare* ATCC 13950 and *M. paraintracellulare* MOTT64, and *M. indicus pranii* MTCC 9506 belong to two different groups, respectively. According to the recent study on genetic comparisons of *M. intracellulare* (12), the two groups are defined as the typical *M. intracellulare* (TMI) group and the *M. paraintracellulare*-*M. indicus pranii* (MP-MIP) group. However, it is noted that only 23.93% (28/117) of the strains were identified as typical *M. intracellulare*, and most strains (76.07%, 89/117) belong to the MP-MIP group, suggesting the strains of the MP-MIP group may be common in clinical isolates in China.

### Phylogenetic analysis

**Figure 1:**
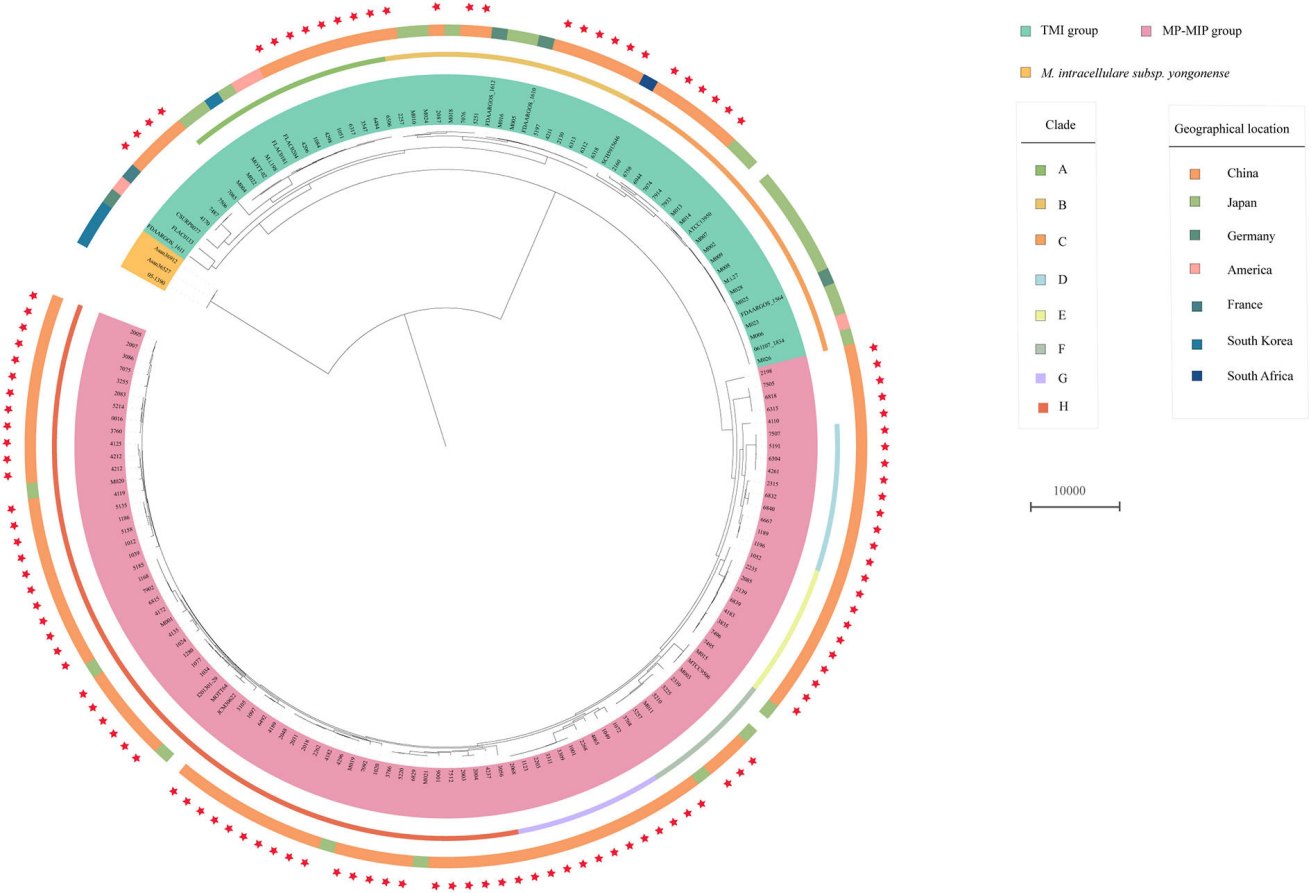
Phylogenetic tree of the 117 clinical *M. intracellulare* strains in this study, 30 *M. intracellulare* strains, and 14 related strains from the NCBI genome database. The tree based on core SNPs was constructed by RAxML with GTR+G model, with 1000 bootstrap replicates. The group type, clade, and geographic location of the strains are shown on the tree (from inner to outer circles), according to the color legend shown on the right. The red stars on the outside represent the strains in this study.

The phylogenetic tree also shows that the 117 strains were divided into two major groups: TMI group and MP-MIP group (Figure 1). The population structure of enrolled strains is in line with the genomic analysis of the *M. intracellulare* in previous reports. In typical *M. intracellulare* (TMI) group, we can see that there are three main clades (clade A, B, C) and every clade, including the strains from different countries, show the clustering of strains was not influenced by the geographical location. Additionally, we discovered that *M. intracellulare* strains had a significant level of genetic diversity, the previously registered strains belong to different clades, such as ATCC13950, M.i.198, and FDAARGOS_1612. In the MP-MIP group, the strains can be classified into five clades (clade D-H), and most strains in our study belong to clade H. The MP reference strain MOTT64 clustered with other 6 strains (1280, 1077, 1034, 1029, 3105, and JCM30622) is in clade H. The MIP reference strain ATCC9506 is a member of clade F, which consists of 9 phylogenetically closely related strains including M003. However, the majority of strains of the MP-MIP group were divided into various clades and clusters, indicating the considerable genetic variability of the genome. Interestingly, we found a public strain of MIP (NFDAARGOS_1610, NZ_CP089222.1) from Germany belonging to the TMI group in our study. By comparing two genome sequences, the ANI identity of this strain with the reference ATCC 13950 (NC_016946.1) and ATCC 9506 (NC_018612.1) is 99.43 and 98.74%, respectively. Thus, this strain may be more related to *M. intracellulare*.

### Antimicrobial susceptibility profiles

**Figure 2:**
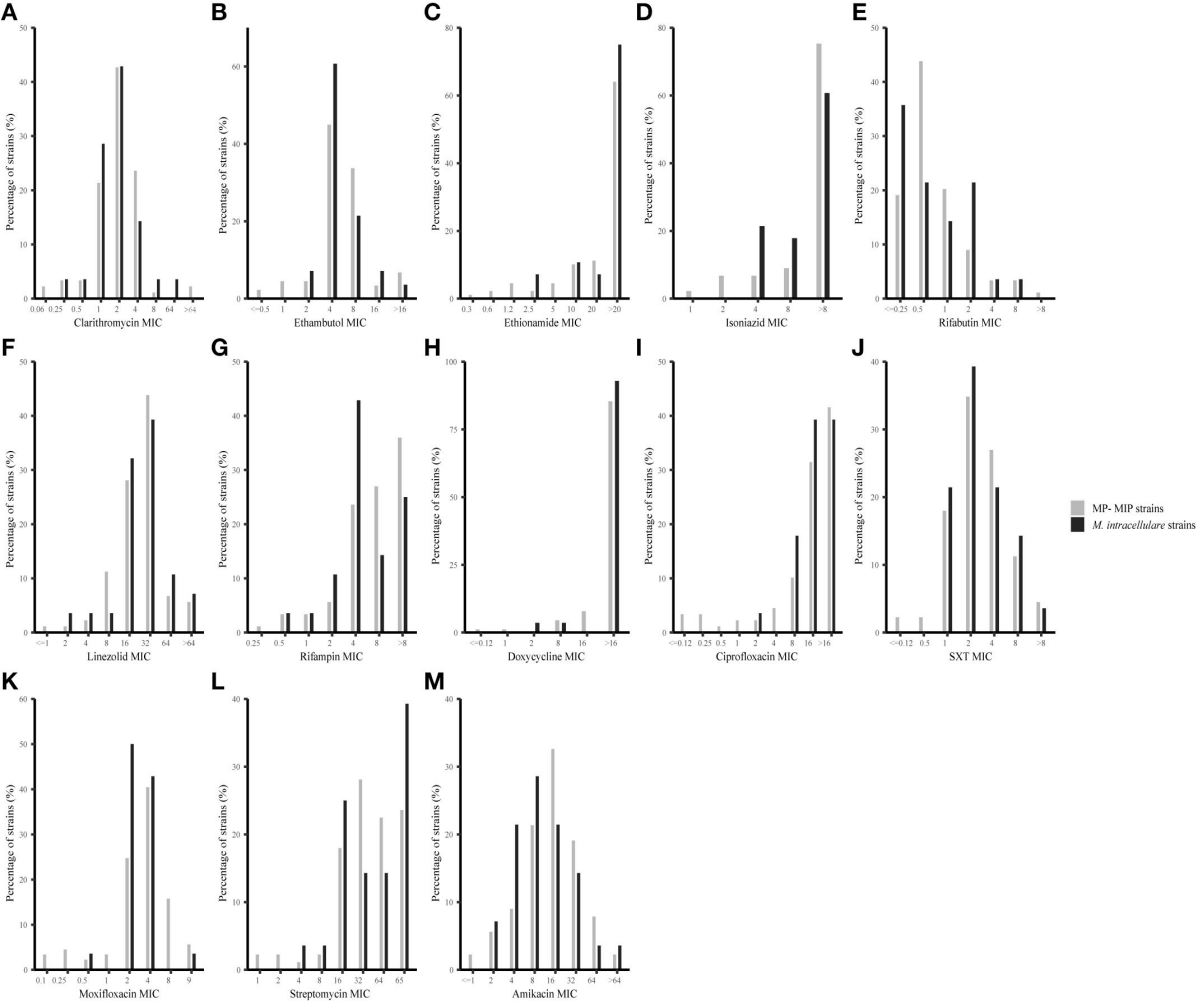
MIC distributions for 28 *M. intracellulare* and 89 MP-MIP strains in this study. The vertical axis of each graph represents the number of strains corresponding to each horizontal coordinate as a percentage of the total number of strains. The black bar and gray bar represent *M. intracellulare and* MP-MIP strains, respectively. **(A–M)** Showed MIC distributions of clarithromycin, ethambutol, ethionamide, isoniazid, rifabutin, linezolid, rifampicin, doxycycline, ciprofloxacin, SXT, moxifloxacin, streptomycin, and amikacin, respectively.

Antimicrobial susceptibilities and MIC range of 28 *M. intracellulare* strains and 89 MP-MIP strains in this study were shown in Table 1 and Figure 2. Clarithromycin was found to be the most effective antibiotic against typical *M. intracellulare* (96.43%) and MP-MIP (97.75%) strains. The MIC_50_ and MIC_90_ were 2 μg/ml and 4 μg/ml, respectively. Amikacin was highly active against the *M. intracellulare* (92.86%) and MP-MIP (89.89%) strains. Rifabutin also shows good activity against the *M. intracellulare* (92.86%) and MP-MIP (92.13%) strains. We also found that most strains are resistant to ciprofloxacin, doxycycline, and rifampicin. The resistant rates of clarithromycin, linezolid, doxycycline, and rifampicin for *M. intracellulare* are higher than MP-MIP strains with no significant difference. However, ethionamide, isoniazid, and streptomycin have no breakpoint established by CLSI, and the MIC_90_ for ethionamide, isoniazid, and streptomycin in this study were >20, >8, and >64 μg/ml, respectively. In addition, we have performed a comparative analysis of drug resistance in different clade strains and have not found a correlation between the clades and drug resistance (Supplementary Figure S2, Supplementary Table S2).

**Table 1:** The antimicrobial susceptibilities and minimum inhibitory concentrations (MICs) of *M. intracellulare* and MP-MIP strains.

	**Critical concentrations** **(**μ**g/ml)**			***M. intracellulare***			**MP-MIP**			***χ^2^***	**P-value**
**Agents**	**S**	**I**	**R**	**MIC50**	**MIC90**	**R (*n*,%)**	**MIC50**	**MIC90**	**R (*n*,%)**		
CLR	8	16	32	2	4	1 (3.57)	2	4	2 (2.25)	–	0.564^*^
AN	16	32	64	8	32	2 (7.14)	16	64	9 (10.11)	0.010	0.922
MXF	1	2	4	2	4	13 (46.43)	4	8	55 (61.80)	2.067	0.151
LNZ	8	16	32	32	64	16 (57.14)	32	64	50 (56.18)	0.008	0.929
CIP	1	2	4	>16	>16	27 (96.43)	>16	>16	78 (87.64)	0.960	0.327
DO	1	2–4	8	>16	>16	27 (96.43)	>16	>16	87 (97.75)	–	0.563^*^
EMB	2	4	8	4	16	9 (32.14)	4	16	39 (43.82)	1.200	0.273
RIF	1		2	4	8	26 (92.86)	8	8	82 (92.13)	0.000	>0.999
RFB	2		4	0.5	2	2 (7.14)	0.5	2	7 (7.87)	0.000	>0.999
SXT	2/38		4/76	2	8	11 (39.29)	2	8	38 (42.70)	0.102	0.750
ETH				>20	>20	-	>20	>20	-		
INH				>8	>8	-	>8	>8	-		
SM				32	>64	-	32	>64	-		

* Indicating *P* value was calculated by Fisher exact test.

### Mutations profiling

In our analysis, the 16S rRNA gene (*rrs*) sequences for amikacin-resistant and amikacin-susceptible strains were identical, which was responsible for amikacin resistance (26). A previous study has described that mutations in 23S rRNA gene (*rrl*) could lead to clarithromycin resistance (27). There are some nucleotide changes in the *rrl* gene, but they are unrelated to drug resistance (Supplementary Table S3). We also investigated the relationship between moxifloxacin resistance and gyrA or gyrB mutation. The peptide sequences of GyrA and GyrB were identical for *M. intracellulare* ATCC 13950 and MIP MTCC 9506, but there were more peptide substitutions in GyrA and GyrB for MP-MIP strains, especially for the GyrB. In comparison to the reference *M. intracellulare* ATCC 13950, 85.39% (76/89) of the MP-MIP isolates had Arg222Lys mutations in gyrA, and 24 and 10 MIP isolates had Glu594Asp and Lys167Gln substitution in GyrB, respectively (Supplementary Table S4), but we have not found any moxifloxacin resistance-associated mutations in GyrA or GyrB, which suggest that mechanisms other than gyrA and gyrB mutations might have contributed to moxifloxacin resistance.

## Discussion

*M. intracellulare* is one of the most common causes of NTM lung disease worldwide, and it has been isolated from clinical pulmonary disease in many areas of China (6, 28). MIP, MP, and *M. intracellulare* are very closely related in the genome. In this study, we analyzed the genome of 117 clinical strains that were previously identified as *M. intracellulare* and presented the phenotypic resistance profile of these strains.

By comparing the genome of clinical *M. intracellulare* strains, we revealed that *M. intracellulare* in China could be classified into two major groups: TMI group and MP-MIP group. This result is supported by a recent report about a genome analysis of *M. intracellulare*, which presented convincing evidence that MP and MIP should be regarded as variants of *M. intracellulare* (12). In our study, 76.07% of strains belong to the MP-MIP group, suggesting that the majority of *M. intracellulare* strains in China could be MP-MIP strains in the genome, and these strains should be considered potential causative agents of pulmonary diseases. With genetic sequencing increasingly affordable, the MP-MIP group strains can be detected more frequently in the future. Though variable numbers of tandem repeats (VNTR) analysis has been a highly discriminatory tool in molecular epidemiology analysis, it is unable to classify the *M. intracellulare* and related strains such as MP and MIP (29). A previous study found that there are 4% of *M. intracellulare* isolates that have been identified as MIP by sequence-based typing analyses (15, 16). Thus, the identification of *M. intracellulare* and related strains should be addressed by multigene sequence analysis or comparative genomic analysis (12, 16). In addition, more research on the pathogenesis of the MP-MIP group is needed, as well as comparisons with *M. intracellulare*.

Our results show that the genetic characteristics of clinical isolates of *M. intracellulare* are not related to geographical location, which is consistent with the previous reports with VNTR analyses (29, 30). In contrast to *M. intracellulare*, the genetic characteristics and molecular epidemiology of clinical strains of the MP-MIP group are poorly understood. Alexander et al. suggested that MIP is a strain of *M. intracellulare* and it is more likely to have specific transposons acquisition and inversion events (31). Our results showed genetic diversity in *M. indicus pranii*, which should be further explored in future studies.

As we all know, few studies reported antimicrobial susceptibility profiles of MP-MIP strains. Our study compared the antimicrobial susceptibilities profile between MP-MIP and *M. intracellulare* strains against 13 drugs, but no statistically significant differences were observed (Table 1). To date, only macrolides have been demonstrated to have a link between *in vitro* susceptibility and clinical responses in patients with MAC lung disease (32). Among the 13 antimicrobials, clarithromycin showed the best activity *in vitro* against *M. intracellulare* isolates, and amikacin has a low resistance rate in our study, which is in line with other studies (33, 34). Rifabutin also has good activity *in vitro* against *M. intracellulare* isolates. Previous studies found that rifabutin is efficacious in multidrug MAC therapy regimens, and it also affects the metabolism and levels of clarithromycin less than rifampin and is generally used to treat disseminated MAC disease (35). Van Ingen et al. did a pharmacokinetic/pharmacodynamic study about the treatment of MAC pulmonary disease, which showed that rifabutin could increase macrolide serum concentrations, especially azithromycin, but rifampin exhibited the opposite (36). Therefore, some experts suggest that rifampin could be replaced with rifabutin in the treatment of MAC infection. The ethambutol and moxifloxacin resistance rates in our strains are much lower than those previously reported in Shanghai, China (20).

Previous studies showed that mutations in the *rrs* and *rrl* genes are associated with aminoglycoside and macrolide resistance, respectively (26, 27). However, none of the tested *M. intracellulare* and MP-MIP strains in our study harbored mutations in the *rrs* genes. This result may be related to the level of drug resistance, as Su-Young Kim et al. showed that mechanisms of high-level resistance to amikacin in MAC isolates involve *rrs* mutations (37). We have not found nucleotide changes in the *rrl* gene related to macrolides resistance, which may be caused by an unknown molecular mechanism. Mutations in gyrA and gyrB are not associated with moxifloxacin resistance in this study, which is consistent with the previous study about *mycobacterium avium* complex isolates (38), suggesting that other mechanisms contribute to moxifloxacin resistance.

A few limitations in this research warrant mention. First, the strains we selected may have sampling bias, resulting in the proportion of the MP-MIP strains being higher than those in previous reports. Therefore, a larger study with more clinical samples of *M. intracellulare* strains is warranted to confirm our findings. Second, this study lacks the clinical background information of the strains, which limited our ability to determine the severity of the disease caused by MP-MIP strains.

## Conclusion

In the present study, we found that clinical *M. intracellulare* strains in China were highly diverse. The phylogenetic analysis found that the *M. intracellulare* strains belong to two major groups: the *M. intracellulare* group and the MP-MIP group, and 76.07% of strains belong to the MP-MIP group. Our finding suggested MP-MIP strains should be considered as a causative agent of severe and chronic lung disease, and its pathogenicity needs to be investigated further. In addition, our data demonstrate no difference in drug susceptibility profiles between *M. intracellulare* and MP-MIP strains. Clarithromycin, amikacin and rifabutin showed strong antimicrobial activity *in vitro* against both *M. intracellulare* and MP-MIP. However, this study showed that there was no clear correlation between the *rrs, rrl*, DNA gyrase genes (*gyrA* and *gyrB*) and the aminoglycosides, macrolides, and moxifloxacin resistance, respectively, indicating other mechanisms might have been involved in drug resistance.

## Data availability statement

The datasets presented in the study are deposited in the NCBI BioProject repository with accession number PRJNA890446.

## Author contributions

ZS, AM, ZL, and YZ contributed to study design, data analysis, and manuscript writing. YW, WH, and DL participated in the study design, data collection, and analysis. PH, CL, XZ, and BZ conducted laboratory testing. HX and SW revised and polished the manuscript. All the authors have read the final version of the manuscript and have approved it.

## Funding

This work was supported by the National Science and Technology Major Project of Infectious Disease (No. 2018ZX10103001).

## Conflict of interest

The authors declare that the research was conducted in the absence of any commercial or financial relationships that could be construed as a potential conflict of interest.

## Publisher's note

All claims expressed in this article are solely those of the authors and do not necessarily represent those of their affiliated organizations, or those of the publisher, the editors and the reviewers. Any product that may be evaluated in this article, or claim that may be made by its manufacturer, is not guaranteed or endorsed by the publisher.

## Supplementary material

Supplementary Figure S1 Pairwise comparison of ANIs of 117 clinical *M. intracellulare* strains in this study. The *M. intracellulare* ATCC 13950, MIP MTCC 9506, *M. intracellulare subsp. yongonense* 05-1390, *Mycobacterium paraintracellulare* MOTT64 and *M. intracellulare subsp. chimaera* DSM 44623 were set as the subspecies reference genomes. The ANI value and the strain type legend are shown on the right.

Supplementary Figure S2 Phylogenetic tree of the 28 *M. intracellulare* strains and 89 MP-MIP strains in this study. The tree based on core SNPs was constructed by RAxML with a GTR model, with 1000 bootstrap replicates. The group type, clades, and drug resistance profile of the strains are shown on the tree (from inner to outer circles), according to the color legend shown on the right.

Supplementary Table S1 The public genomes of 30 *M. intracellulare* and 14 related strains were used in this study.

Supplementary Table S2 The antimicrobial susceptibilities and minimum inhibitory concentrations (MICs) of strains in different clades.

Supplementary Table S3 The 23S RNA mutation of 28 *M. intracellulare* and 89 MP-MIP strains in this study.

Supplementary Table S4 The GyrA and GyrB mutation of 28 *M. intracellulare* and 89 MP-MIP strains in this study.
