# Supplementary figures and images for: High diversity of clinical Mycobacterium intracellulare in China revealed by whole genome sequencing
Source: Front Public Health. 2022 Nov 17;10:989587. doi: 10.3389/fpubh.2022.989587 (PMC9714602; doi:10.3389/fpubh.2022.989587)

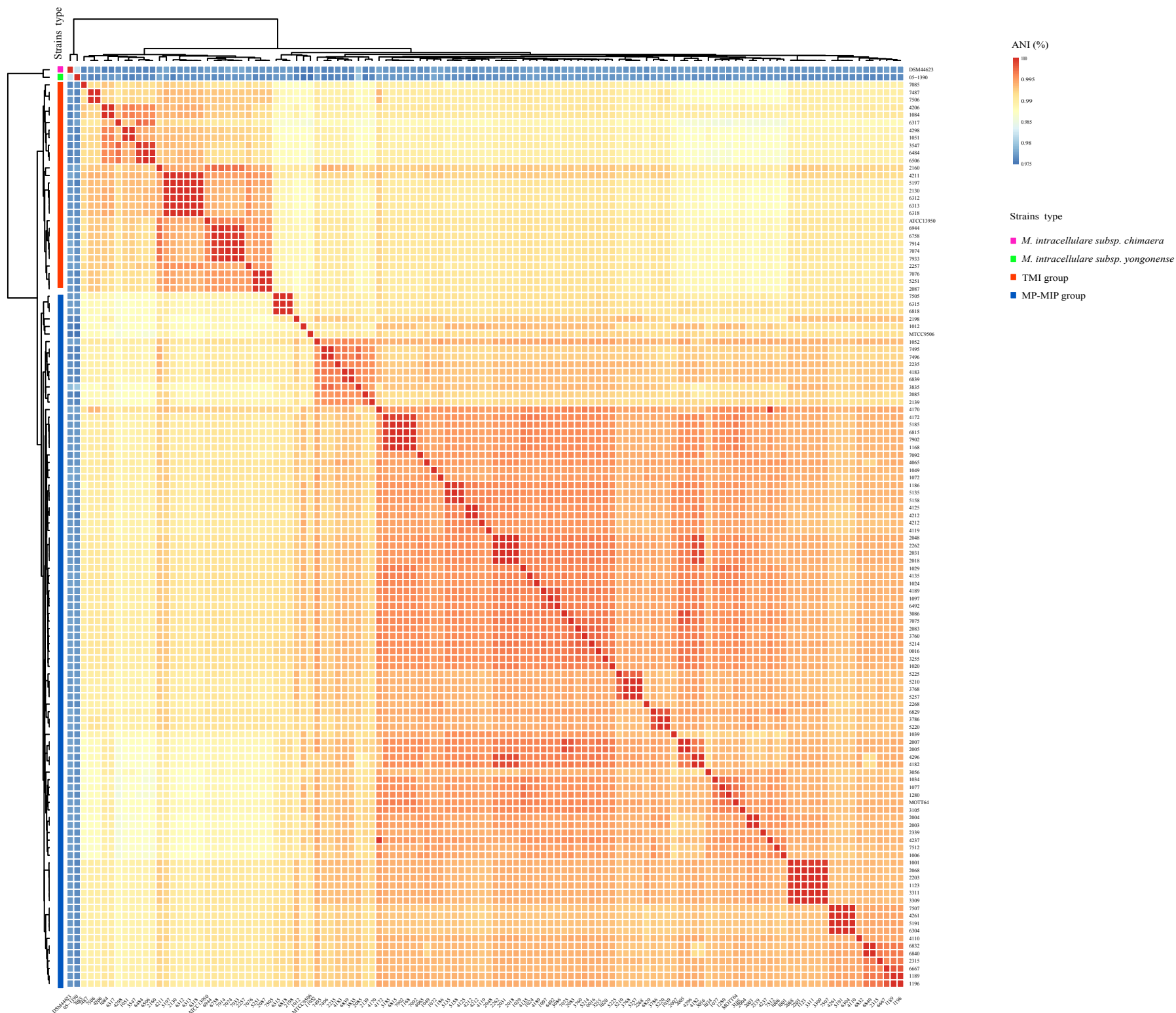

Supplement: Supplementary Figure S1 — Pairwise comparison of ANIs of 117 clinical M. intracellulare strains in this study. The M. intracellulare ATCC 13950, MIP MTCC 9506, M. intracellulare subsp. yongonense 05-1390, Mycobacterium paraintracellulare MOTT64 and M. intracellulare subsp. chimaera DSM 44623 were set as the subspecies reference genomes. The ANI value and the strain type legend are shown on the right. [file Data_Sheet_1.PDF]

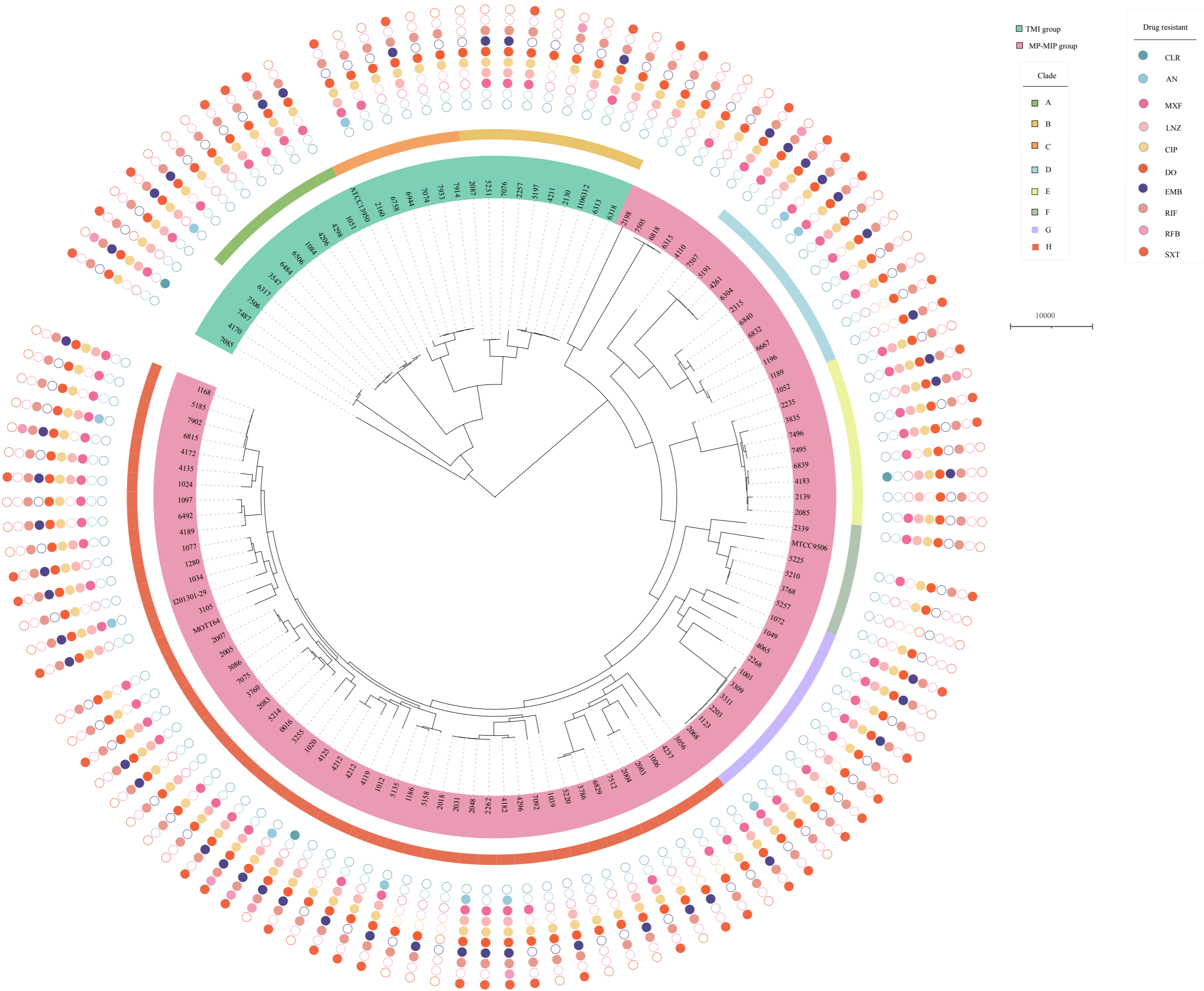

Supplement: Supplementary Figure S2 — Phylogenetic tree of the 28 M. intracellulare strains and 89 MP-MIP strains in this study. The tree based on core SNPs was constructed by RAxML with a GTR model, with 1000 bootstrap replicates. The group type, clades, and drug resistance profile of the strains are shown on the tree (from inner to outer circles), according to the color legend shown on the right. [file Data_Sheet_2.PDF]
